# Simultaneous Detection and Differentiation of Highly Virulent and Classical Chinese-Type Isolation of PRRSV by Real-Time RT-PCR

**DOI:** 10.1155/2014/809656

**Published:** 2014-06-12

**Authors:** Shuqi Xiao, Yaosheng Chen, Liangliang Wang, Jintao Gao, Delin Mo, Zuyong He, Xiaohong Liu

**Affiliations:** State Key Laboratory of Biocontrol, School of Life Sciences, Sun Yat-sen University, Guangzhou 510006, China

## Abstract

Porcine reproductive and respiratory syndrome (PRRS) is a leading disease in pig industry worldwide and can result in serious economic losses each year. The PRRS epidemic situation in China has been very complicated since the unprecedented large-scale highly pathogenic PRRS (HP-PRRS) outbreaks in 2006. And now the HP-PRRS virus (HP-PRRSV) and classical North American type PRRSV strains have coexisted in China. Rapid differential detection of the two strains of PRRSV is very important for effective PRRS control. The real-time RT-PCR for simultaneous detection and differentiation of HP-PRRSV and PRRSV by using both SYBR Green and TaqMan probes was developed and validated. Both assays can be used for rapid detection and strain-specific identification of HP-PRRSV and PRRSV. However, the TaqMan probe method had the highest detection rate whereas the conventional RT-PCR was the lowest. The real-time RT-PCR developed based on SYBR Green and TaqMan probe could be used for simultaneous detection and differentiation of HP-PRRSV and PRRSV in China, which will benefit much the PRRS control and research.

## 1. Introduction

Porcine reproductive and respiratory syndrome (PRRS) is widely accepted as being one of the most economically important diseases affecting swine industry [1]. In 2006 there was an unparalleled large-scale outbreak of the so-called high fever disease in most provinces of China that affected more than 2,000,000 pigs, leading to concerns within the global swine industry and in relation to public health [2–4]. In March 2007 the disease was identified in the Hai Duong province of Vietnam and it spread countrywide affecting more than 65,000 pigs [5, 6]. The outbreaks caused extensive concern worldwide [7]. Studies demonstrated that highly virulent porcine reproductive and respiratory syndrome virus (HP-PRRSV) was the major causative pathogen of the so-called high fever disease [2]. Genetic analysis indicated that the HP-PRRSVs isolated from China and Vietnam shared a discontinuous deletion of 30 aa in nonstructural protein 2 (NSP2), as compared with the North American type PRRSV strains (NA PRRSV) [2, 5, 8]. Since 2006, the HP-PRRSV and classical North American type PRRSV strains coexist in China. Now PRRS epidemic situation is very complicated in China, of which the predominant form is the HP-PRRSV. Rapid differential detection of the two strains of PRRSV is very important for effective PRRS control. Therefore, it is imperative to develop an assay for simultaneous detection and strain identification of HP-PRRSV and PRRSV.

The current immunoassay, such as immunohistochemistry and serological methods, cannot differentiate between the two strains of PRRSV. Conventional RT-PCR is time-consuming, lowly sensitive, and also prone to contamination. The development of real-time RT-PCR technology offers the opportunity for more rapid, sensitive, and specific detection of virus. The current two major genotypes, the European (EU) and the North American (US) strains, have been rapidly identified by SYBR Green-based or TaqMan probe-based real-time RT-PCR assay [9–11]. A specific TaqMan probe real-time RT-PCR has been developed for assaying the HP-PRRSV [12], but it is not able to differentially detect the HP-PRRSV and PRRSV.

In this research, the real-time RT-PCR for simultaneous detection and differentiation of HP-PRRSV and PRRSV by using both SYBR Green and TaqMan probe was developed and validated. These two methods provided alternative diagnostic assays in diverse PRRSV epidemiological circumstances.

## 2. Materials and Methods

### 2.1. Virus Strains and Clinical Samples

HP-PRRSV (GD and XH) and PRRSV (CH-1a) virus strains were kindly supplied by Dr. Guihong Zhang (South China Agricultural University, China). PRRSV (CC), PRV, FPV, and FCV were kindly provided by Laboratory Animal Center in Jilin University, China. 39 and 477 serum samples were obtained from 6 pig farms in South China in 2008 and 2011, respectively. 15 sera as described previously were from pigs experimentally infected with HP-PRRSV and PRRSV [13]. The viral RNA of the virus-infected cell culture and serum was extracted by using QIAamp Viral RNA Mini Kit according to the manufacturer's instruction (Qiagen). First-strand cDNA was synthesized using the extracted total RNA and AMV Reverse Transcriptase from Reverse Transcription System of Promega according to the manufacturer's instruction (Promega).

### 2.2. PCR Primers and Probes

The difference of genome sequence between the HP-PRRSV and PRRSV was the 87-base deletion in the fixed site in NSP2 gene [2, 12]. After aligning 20 HP-PRRSV and PRRSV strains isolated from China and the US strain (VR-2332) sequences obtained from the NCBI database, the NSP2 region was selected to design an assay for discriminating between HP-PRRSV and PRRSV strains. The differential detection based on real-time RT-PCR using SYBR Green I and TaqMan probes was performed employing the same primer pair (Table 1). Real-time RT-PCR for PRRSV detection based on dual-colour TaqMan probes was performed using strain-specific probes including a Pb-H (only detecting HP-PRRSV strain) [12], Pb-N (only detecting PRRSV strain), and Pb-all (simultaneously detecting both HP-PRRSV and PRRSV strains) (Table 1).

SYBR Green I real-time PCR was carried out using SYBR Premix Ex Taq (TaKaRa) and the LightCycler 480 Real-Time PCR System (Roche Applied Science). Amplification was performed in a 10 *μ*L reaction mixture containing 5.0 *μ*L SYBR Premix Ex Taq (2×), 0.2 *μ*L of each forward (NSP2-qF) and reverse (NSP2-qR) primer (10*μ*M), 1.5 *μ*L cDNA or plasmid DNA, and 3.1 *μ*L H2O. The amplification conditions were 95°C for 10 s, followed by 40 cycles of 95°C for 5 s and 60°C for 40 s. Fluorescent signal was detected for each cycle at the end of the 60°C extension step. For each assay, a standard curve was generated with 10-fold serially diluted plasmid standards of 10^2^–10^6^ copies/*μ*L. Meanwhile positive and negative reference samples were detected along with unknown samples. After 40 amplification cycles, melting curve analysis was carried out with the conditions of 95°C for 1s and 60°C for 15 s and then increased to 95°C while continuously collecting the fluorescent signal. The melting temperature (Tm) of each strain was analyzed to verify the PRRSV type.

The 10 *μ*L duplex TaqMan probe real-time PCR reaction mixtures contained 5.0 *μ*L Premix Ex Taq (2×) (TaKaRa), 0.2 *μ*L of each forward (NSP2-qF) and reverse (NSP2-qR) primer (10 *μ*M), 0.2 *μ*L of each probe (Pb-H and Pb-N or Pb-all and Pb-N, 10 *μ*M), 1.5 *μ*L cDNA or plasmid DNA, and 2.7 *μ*L H_2_O. The amplification conditions were 95°C for 10 s, followed by 45 cycles of 95°C for 5 s and 60°C for 40 s. For each assay, a standard curve was generated with 10-fold serially diluted plasmid standards of 10^1^–10^6^ copies/*μ*L. The FAM (6-carboxyfluorescein) and HEX (hexachloro-6-carboxyfluorescein) signals were detected for each cycle at the end of the 60°C extension step.

### 2.3. Conventional RT-PCR and Preparation of Standard Plasmid DNA

The conventional RT-PCR was performed by using the NSP2-F and NSP2-R primers described in Table 1. 10 *μ*L reaction mixture contains 0.5 *μ*L cDNA, 5.0 *μ*L 2 × PCR reaction mix, 0.4 *μ*L NSP2-F (10 *μ*M) primer, 0.4 *μ*L NSP2-R (10 *μ*M) primer, 0.1 *μ*L Taq DNA polymerase (2.5 U/*μ*L), and 3.6 *μ*L H_2_O. The negative controls included the reagents without cDNA template. The reaction mixtures were performed at the amplification condition: 95°C for 3 min, followed by 30 cycles of 94°C for 30 s, 60°C for 30 s, and 72°C for 1 min, and a final extension step of 5 min at 72°C. The PCR products were detected by 1.5% agarose gel electrophoresis in 1 × TAE. Then the PCR products were cloned into the plasmid pMD20-T (TaKaRa) and propagated in competent* Escherichia coli* DH5*α* cells according to the manufacturer's instructions. Plasmid DNA was purified using the E.Z.N.A. Plasmid Mini Kit I (Omega) and quantified by measuring OD_260_ using spectrophotometer ND-1000 (Wilmington, USA).

## 3. Results and Discussion

### 3.1. SYBR Green I Real-Time PCR

**Figure 1:**
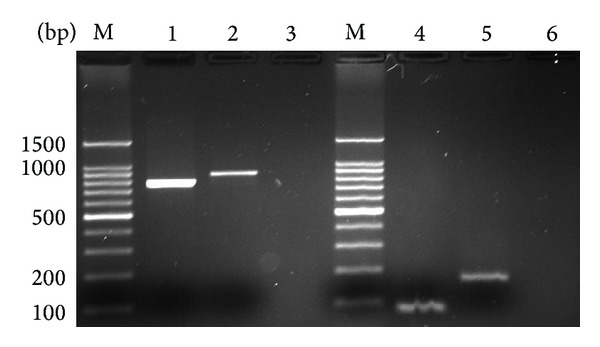
Conventional PCR results of PRRSV NSP2 gene. M: 100 bp marker; 1 and 4: HP-PRRSV (GD) strain; 2 and 5: PRRSV (CH-1a) strain; 3 and 6: negative.

**Figure 2:**
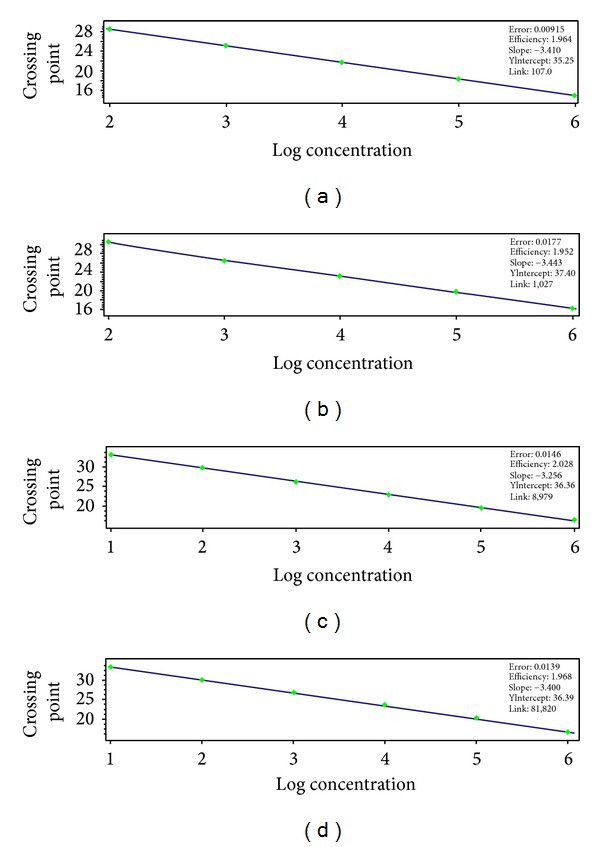
Standard curves were generated based on Cp values of 10-fold dilutions of plasmid DNA. Regression lines between the Cp (*C*_*T*_) values and the input concentrations of HP-PRRSV (a) and PRRSV (b) plasmid DNA in real-time RT-PCR detected using SYBR Green I and HP-PRRSV (c) and PRRSV (d) using TaqMan probe, respectively.

**Figure 3:**
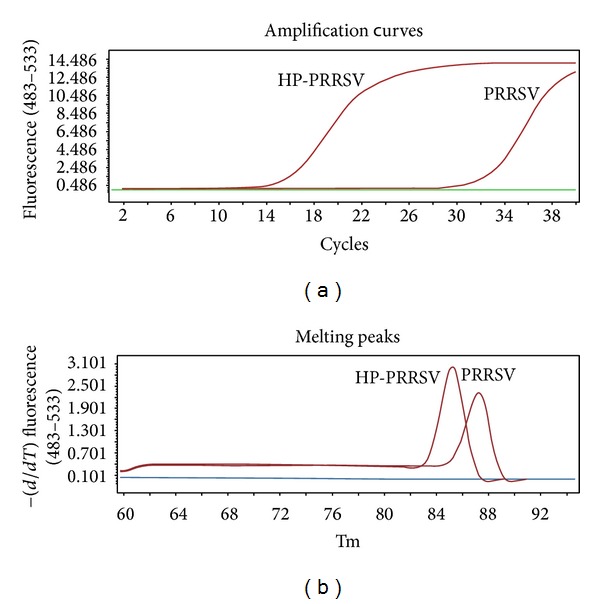
Specific amplification curves and melting curve analysis by SYBR Green I real-time PCR. (a) Specific amplification curves. Fluorescent curves were observed when HP-PRRSV (GD) and PRRSV (CH-1a) were used as templates; no fluorescent signals were observed when the templates were other viruses and host cells. (b) Melting curves. Tm of HP-PRRSV = 85.17 ± 0.12°C; Tm of PRRSV = 87.27 ± 0.07°C.

10-fold serial plasmid dilutions were tested and used to construct the standard curve. The generated standard curve covered a linear range of 3.93 × 10^2^ to 3.93 × 10^6^ copies/*μ*L for HP-PRRSV and 8.56 × 10^2^ to 8.56 × 10^6^ copies/*μ*L for PRRSV. Both standard curves had a slope of −3.410 to −3.443 and an efficiency of 1.964 to 1.952, which indicate a high PCR efficiency of the experiment (Figures 2(a) and 2(b)). The amplification with primers NSP2-qF and NSP2-qR yielded 85 bp and 172 bp amplified product within NSP2 of both HP-PRRSV (GD) and PRRSV (CH-1a), respectively (Figure 1), which was sufficient to discriminate between melting peaks of the two PRRSV strains. The mean and standard deviation of Tm of HP-PRRSV and PRRSV were 85.17 ± 0.12°C and 87.27 ± 0.07°C, respectively (Figure 3(b)).

### 3.2. TaqMan Probe Real-Time PCR

**Figure 4:**
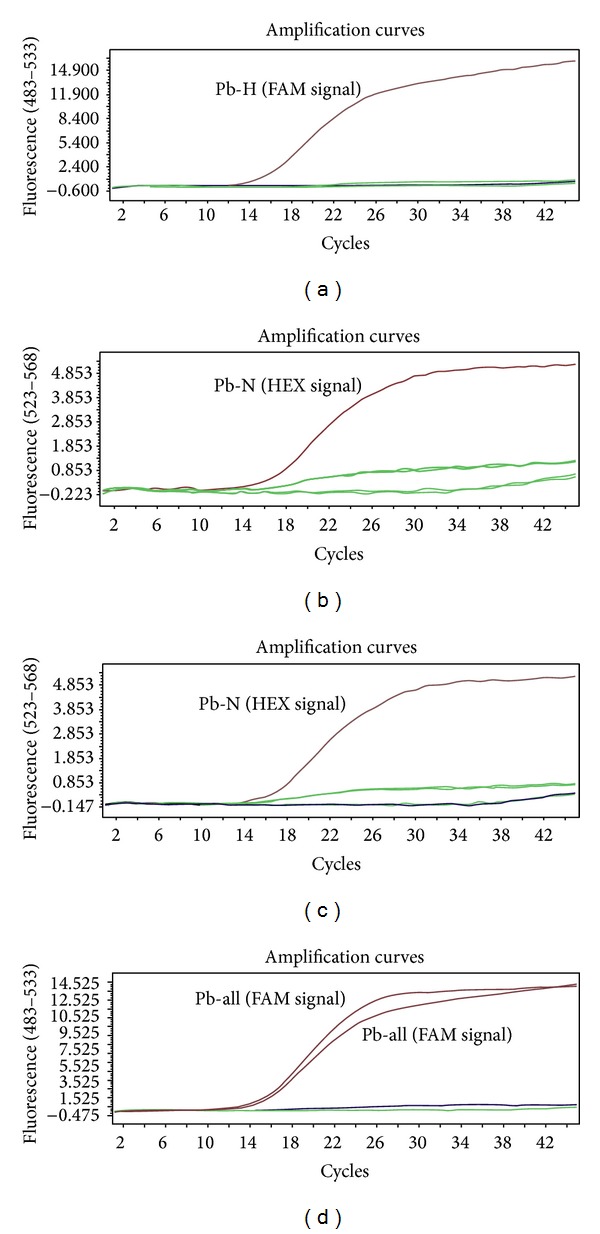
Specific amplification curves by duplex TaqMan probe real-time PCR. When Pb-H (FAM) and Pb-N (HEX) probes were combined in a duplex real-time PCR system, only the FAM fluorescent signal could be observed when the template was GD HP-PRRSV strain, no FAM signal was detected when the templates were CH-1a PRRSV strain and other viruses (a), and vice versa, only the HEX signal could be collected when the template was CH-1a PRRSV strain (b). When Pb-N (HEX) and Pb-all (FAM) were combined in a duplex real-time PCR system, the Pb-N (HEX signal) probe could only detect PRRSV strain (c), whereas Pb-all (FAM signal) probe could detect both HP-PRRSV and PRRSV strains (d).

The generated standard curve covered a linear range of 3.93 × 10^1^ to 3.93 × 10^6^ copies/*μ*L for HP-PRRSV and 8.56 × 10^1^ to 8.56 × 10^6^ copies/*μ*L for PRRSV. Both standard curves had a slope of −3.256 to −3.400 and an efficiency of 2.028 to 1.968, which indicate a high PCR efficiency of the experiment (Figures 2(c) and 2(d)). Two TaqMan probes specific to HP-PRRSV and PRRSV strains combined in duplex real-time PCR system can specifically detect the two PRRSV strains. When the two TaqMan probes of Pb-H (FAM) and Pb-N (HEX) were combined in a duplex real-time PCR system, only the FAM fluorescent signal could be observed in the template of GD HP-PRRSV strain, and only the HEX fluorescent signal could be observed in the template of CH-1a PRRSV strain (Figure 4). However, when Pb-N (HEX) and Pb-all (FAM) were combined in a duplex real-time PCR system, only HEX fluorescent signal could be observed when the template was CH-1a PRRSV strain, and FAM fluorescent signal could be observed when the templates were GD and CH-1a strains (Figure 4).

### 3.3. Validation of Real-Time PCR Assay

Specificity of real-time PCR using SYBR Green I and TaqMan probe was determined by analyzing nucleic acid extracts of other viruses (PRV, FPV, and FCV), host cells (Marc145, PK15), and H_2_O. The results of the specificity test of the two methods showed that there were no cross-amplifications from other viruses or host cells (Figures 3(a) and 4), which confirmed that the primers and probes used in this study were highly specific for both HP-PRRSV and PRRSV.

**Figure 5:**
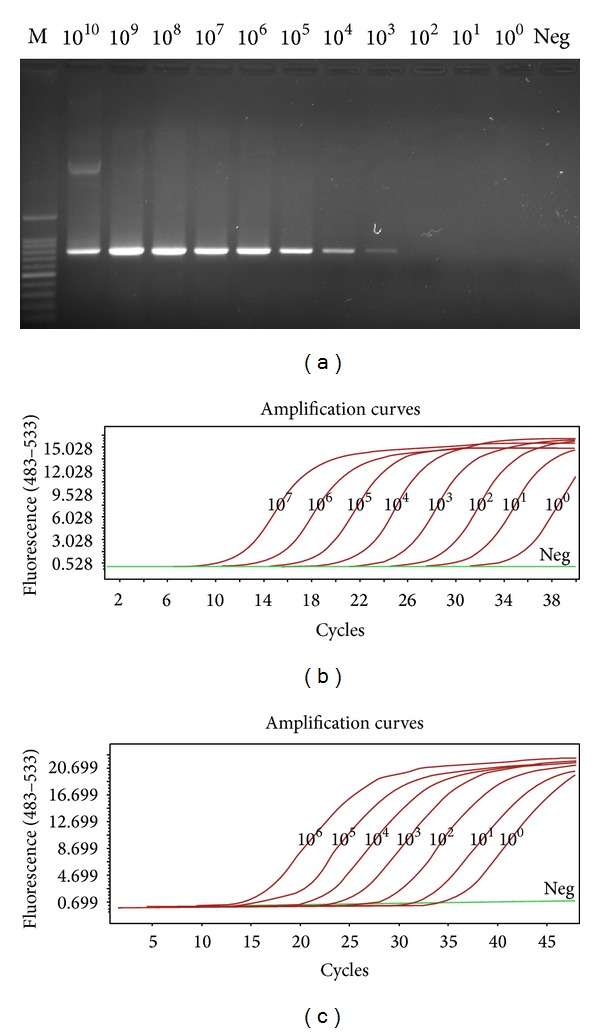
Comparison of sensitivity for HP-PRRSV detection by conventional RT-PCR and real-time PCR. Samples were 10-fold serially diluted plasmid standards of HP-PRRSV. M: 100 bp marker; Neg: negative control.

**Figure 6:**
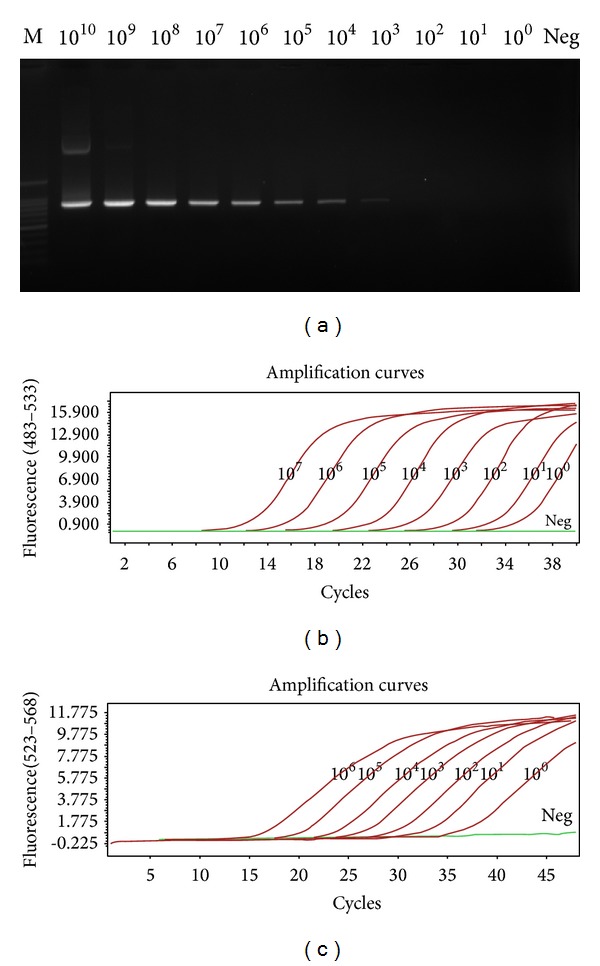
Comparison of sensitivity for PRRSV detection by conventional RT-PCR and real-time PCR. Samples were 10-fold serially diluted plasmid standards of PRRSV. M: 100 bp marker; Neg: negative control.

10-fold serially diluted plasmid standards of HP-PRRSV (pMD20-GD) and PRRSV (pMD20-CH1a) were used as templates for sensitivity tests in both conventional PCR and real-time PCR using SYBR Green I and TaqMan probe. The results showed that real-time PCR using both SYBR Green I (Figure 6) and TaqMan probe (Figure 5) can be used to detect concentrations at least 10^0^ copies/*μ*L of plasmid standards whereas the sensitivity of conventional PCR was only 10^3^ copies/*μ*L.

The intra- and interassay reproducibility were evaluated using three replicates of 10^6^, 10^4^, and 10^2^ copies/*μ*L plasmid standards of both pMD20-GD and pMD20-CH1a. Mean and coefficient of variation (CV) for the *C*_*T*_ value were calculated. The results showed that neither the CVs of intra-assay nor the CVs of interassay were more than 5% (Table 2), indicating the reproducibility of the two assays.

Our results showed that real-time PCR using both SYBR Green I and TaqMan probe could be used to simultaneously detect and differentiate HP-PRRSV and PRRSV in China. But the TaqMan probe method had the highest detection rate, whereas the conventional RT-PCR was the lowest. The SYBR Green I real-time PCR assay is timesaving, easy to handle, and highly sensitive. Yang et al. detected the PRRSV and CSFV RNA by SYBR Green I-based quantitative PCR and found that both sensitivity and specificity were equal or superior to conventional RT-PCR [14]. Although Tian et al. developed a rapid SYBR one step real-time RT-PCR for detection of PRRSV [15], it could not be used for simultaneous detection and differentiation of HP-PRRSV and classical North American type PRRSV (PRRSV). Kleiboeker et al. developed dual labeled probes quantitative PCR, which could simultaneously detect NA- and EU-PRRSV [16]. However, this assay could not simultaneously detect and differentiate between both HP-PRRSV and classical North American type PRRSV (PRRSV) strains in China. The TaqMan probe method provided more accurate results than SYBR Green I with melting curve analysis. SYBR Green I real-time PCR assay was simpler, rapider, and lower in cost than TaqMan probe method. In addition to the high specificity, sensitivity, and reproducibility, the real-time PCR assay based on both SYBR Green I and TaqMan probe established by us could recognize coinfection of HP-PRRSV and PRRSV. Because the two types of PRRSV isolates coexist in Chinese swine herds, recombination could occur. Therefore, the results provided alternative diagnostic assays in diverse PRRSV epidemiological circumstances.

### 3.4. Testing of Clinical Samples

To compare and evaluate the developed real-time RT-PCR and conventional RT-PCR, 2 reference strains of H-PRRSV (GD and XH) and N-PRRSV (CH-1a and CC) and 15, 39, and 477 serum samples were tested. The results were shown in Table 3. The results of 4 reference strains for real-time PCR assays were consistent with that of conventional RT-PCR method. The results of 531 serum samples showed that the TaqMan probe real-time PCR had the highest detection rate, whereas the conventional RT-PCR had the lowest detection rate. To evaluate comprehensively the practicality of this assay, clinical samples that span a broader geographical origin should be tested in the future.

## 4. Conclusions

The real-time RT-PCR for simultaneous detection and differentiation of HP-PRRSV and PRRSV by using both SYBR Green and TaqMan probes was developed and validated. Both assays can be used for rapid detection and strain-specific identification of HP-PRRSV and PRRSV. A total of 535 samples were tested by real-time PCR and conventional RT-PCR. The results of 4 reference strains for real-time PCR assays were consistent with that of conventional PCR method. The results of 531 serum samples showed that the TaqMan probe method had the highest detection rate whereas the conventional RT-PCR was the lowest. The real-time PCR developed based on SYBR Green and TaqMan probe could be used for simultaneous detection and differentiation of HP-PRRSV and PRRSV in China, which provided two alternative diagnostic assays in diverse PRRSV epidemiological circumstances.

## Figures and Tables

**Table 1:** Primers and probes used in the real-time RT-PCR and conventional RT-PCR assays.

Primers and probes		Sequences (5′-3′)	Products (bp)	
			HP-PRRSV	PRRSV
Conventional RT-PCR	NSP2-F	AACACCCAGGCGACTTCA	787	874
	NSP2-R	GCATGTCAACCCTATCCCAC		

Real-time RT-PCR	NSP2-qF	GTGGGTCGGCACCAGTT	85	172
	NSP2-qR	GACGCAGACAAATCCAGAGG		

Probes	Pb-H	FAM-CGCGTAGAACTGTGACAACAACGCTGA-TAMRA [12]		
	Pb-N	HEX-AAAATTGGCTCACTCAAGGGCGTCA-TAMRA		
	Pb-all	FAM-CACAGTTCTACGCGGTGCAGG-TAMRA		

**Table 2:** Intra- and interassay reproducibility of real-time PCR.

Concentration of standard plasmid (copies/*μ*L)	*n*	Intra-assay (Cp)			Interassay (Cp)		
		Mean	SD	CV (%)	Mean	SD	CV (%)
HP-PRRSV (SYBR)							
10^6^	3	14.96	0.02	0.13	14.82	0.29	1.96
10^4^	3	21.78	0.04	0.18	21.49	0.57	2.65
10^2^	3	28.5	0.16	0.56	28.48	0.27	0.95
PRRSV (SYBR)							
10^6^	3	15.47	0.02	0.13	15.75	0.31	1.97
10^4^	3	22.55	0.05	0.22	22.71	0.19	0.84
10^2^	3	29.6	0.01	0.03	29.96	0.34	1.13
HP-PRRSV (FAM)							
10^6^	3	15.9	0.02	0.13	15.98	0.06	0.38
10^4^	3	22.51	0.01	0.04	22.63	0.15	0.66
10^2^	3	29.71	0.02	0.07	29.59	0.17	0.57
PRRSV (HEX)							
10^6^	3	16.59	0.15	0.90	16.47	0.04	0.24
10^4^	3	23.41	0.09	0.38	23.44	0.37	1.58
10^2^	3	29.75	0.04	0.13	29.56	0.28	0.95

**Table 3:** Detection results of samples by conventional and real-time PCR.

Samples	Number	Methods					
		Conventional PCR		SYBR Green I		TaqMan probe	
		HP-PRRSV	PRRSV	HP-PRRSV	PRRSV	HP-PRRSV	PRRSV
Reference strains	4	2	2	2	2	2	2
Serum 1	15	6	4	6	5	6	6
Serum 2	39	0	5	0	6	0	8
Serum 3	477	2	0	19	0	34	7
